# Environmental systems biology of cold-tolerant phenotype in *Saccharomyces* species adapted to grow at different temperatures

**DOI:** 10.1111/mec.12930

**Published:** 2014-10-21

**Authors:** Caroline Mary Paget, Jean-Marc Schwartz, Daniela Delneri

**Affiliations:** Faculty of Life Sciences, Michael Smith Building, University of ManchesterManchester, M13 9PT, UK

**Keywords:** adaptation, *Saccharomyces kudriavzevii*, systems biology, temperature, thermodynamics

## Abstract

Temperature is one of the leading factors that drive adaptation of organisms and ecosystems. Remarkably, many closely related species share the same habitat because of their different temporal or micro-spatial thermal adaptation. In this study, we seek to find the underlying molecular mechanisms of the cold-tolerant phenotype of closely related yeast species adapted to grow at different temperatures, namely *S. kudriavzevii* CA111 (cryo-tolerant) and *S. cerevisiae* 96.2 (thermo-tolerant). Using two different systems approaches, *i*. thermodynamic-based analysis of a genome-scale metabolic model of *S. cerevisiae* and *ii*. large-scale competition experiment of the yeast heterozygote mutant collection, genes and pathways important for the growth at low temperature were identified. In particular, defects in lipid metabolism, oxidoreductase and vitamin pathways affected yeast fitness at cold. Combining the data from both studies, a list of candidate genes was generated and mutants for two predicted cold-favouring genes, *GUT2* and *ADH3*, were created in two natural isolates. Compared with the parental strains, these mutants showed lower fitness at cold temperatures, with *S. kudriavzevii* displaying the strongest defect. Strikingly, in *S. kudriavzevii,* these mutations also significantly improve the growth at warm temperatures. In addition, overexpression of *ADH3* in *S. cerevisiae* increased its fitness at cold. These results suggest that temperature-induced redox imbalances could be compensated by increased glycerol accumulation or production of cytosolic acetaldehyde through the deletion of *GUT2* or *ADH3*, respectively.

## Introduction

Environmental change is a key driving force for the adaptation of species. However, we are a long way from understanding the effects of specific species–environment interactions on the evolution of organisms and ecological systems. Temperature has a major influence on the activity and performance of ecosystems and has been identified as one of two predominant factors governing biomass production, with the other being body size (Enquist *et al*. 2003). There are many examples in nature where closely related species coexist in the same environmental niche due to thermo-adaptation. Examples include, but are not limited to, the temporal difference in the development of *Ambystoma* salamander larvae in Florida (Keen *et al*. 1984); depth and rate of burrowing of *Laternula* bivalves in Singaporean mangroves (Lai *et al*. 2011) and the foraging schedules of *Myrmecia* ants in Australia (Jayatilaka *et al*. 2011). In each of these cases, it has been hypothesized that the different species have adapted to a specific temperature to avoid competition with the closely related species. The bivalves and the ants have adapted to circadian temperature cycles and the salamander larvae to seasonal temperatures.

The overall effect of environmental temperature on an organism phenotype has not been explicitly linked to a precise intracellular biochemical or metabolic process. In a complex biological network, a systems biology approach may help to identify key pathways and functional modules related to a particular phenotype, helping to advance towards a predictive model of biological processes (Woolf *et al*. 2010). Currently, computational researchers have used temperature-based modelling techniques to study the feasibility and directionality of biochemical reactions (Henry *et al*. 2006), but no effort has been carried out to associate temperature fluctuations to adaptation processes in vivo.

Yeast represents an established experimental system for which many genome-scale metabolic models have been constructed, mostly for *Saccharomyces cerevisiae* (Duarte *et al*. 2004; Herrgard *et al*. 2008; Mo *et al*. 2009). *Saccharomyces cerevisiae* is the most widely studied member of the *Saccharomyces* clade which also inclu-des *S. paradoxus, S. cariocanus*, *S. mikatae, S. kudriavzevii, S. arboriculus, S. uvarum, S. eubayanus, S. bayanus and S. pastorianus*. This sister group have high genetic similarity and are ubiquitous, but display different phenotypes (Sampaio & Goncalves 2008; Arroyo-Lopez *et al*. 2009). For example, *S. cerevisiae*, *S. paradoxus* and *S. mikatae* grow at an optimal temperature of 30 °C, while *S. uvarum* and *S. pastorianus*, species associated with wine and larger fermentation processes respectively, are considered cryo-tolerant, growing well at low temperatures of 10–12 °C. It has been shown that, across a number of isolates from the same *Saccharomyces* species, the optimal, maximal and minimal growth temperatures were consistent (Salvadó *et al*. 2011).

A study of the distribution of the yeast population on Mediterranean oaks has also established the co-occurrence on these trees of both *S. cerevisiae* and the cold-tolerant species *S. kudriavzevii* (Sampaio & Goncalves 2008). This sympatric association is likely to be caused by different growth temperature preferences of the two yeast species, with *S. kudriavzevii* better adapted to cold conditions. In fact, the average optimal growth temperature for *S. cerevisiae* is 32 °C, whereas *S. kudriavzevii* displays an optimal fitness around 24 °C (Salvadó *et al*. 2011).

These yeast isolates are a good experimental model for studying temperature-dependent phenotype as they live in sympatry (i.e. ecological niche is kept constant), but display a different thermo-growth profile. It is therefore possible to discriminate and compare pathways and genes involved in the cold tolerance trait. Several studies have integrated computational modelling with yeast experimental data at the genome scale (King *et al*. 2004; Schwartz *et al*. 2007; Feist *et al*. 2010; Smallbone *et al*. 2010), and other literature is available on the inclusion of thermodynamics, although mostly in bacteria (Beard *et al*. 2002; Henry *et al*. 2007; Fleming *et al*. 2010; Jol *et al*. 2012). However, none of these models currently include thermodynamic data and growth comparisons at different temperatures.

**Figure 1:**
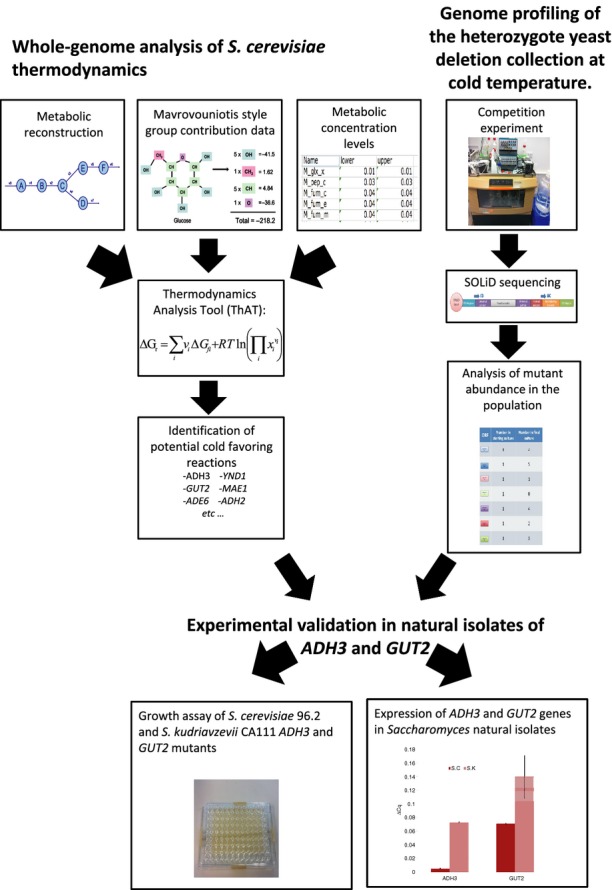
A flowchart of the strategy used in this study. Starting with a list of ΔG of formation of metabolites calculated using a Mavrovouniotis style group contribution method and a range of metabolite concentrations, we analysed the reactions within the *i*MM904 *Saccharomyces cerevisiae* metabolic model for their thermodynamic properties. We also carried out completion experiments of the yeast deletion collection in three different media types and calculated the change in mutant levels through SOLiD sequencing. The combination of these experiments led to a list of predicted cold-favouring reaction genes that can be verified in natural isolates.

In this study, a systems biology approach has been taken to identify the genetic mechanisms underlying the cold phenotype in yeast species, adapted to grow at different temperatures. We have widened the scope of metabolic-coupled thermodynamic analysis by testing how the free energy of the reactions within a *S. cerevisiae* metabolic model changes with temperature. Also, a genome-scale yeast deletion collection competition experiment at low temperature was carried out to find processes that aid cold acclimation (Fig.1). Based on these large-scale studies, we selected two candidate genes, *ADH3* and *GUT2*, to investigate their phenotype in *Saccharomyces cerevisiae* 96.2 and *Saccharomyces kudriavzevii* CA111 strains, which have optimal growth temperatures of 32 and 24 °C, respectively. Knockout and expression studies carried out in these yeasts revealed significant fitness changes in the thermo-growth profiles, indicating the disrupted genes are important for cold tolerance in these natural isolates.

Overall, this study integrates genomewide screening, thermodynamic analyses and experimental validations in natural yeast species to understand cold tolerance. This represents the first effort to study temperature-dependent phenotypes in a systems biology fashion and to extrapolate laboratory settings to a natural system in an attempt to address both the need to perform rigorously controlled experiments and to tackle ecologically relevant issues.

## Materials and methods

### Thermodynamic analysis tool

A Java program was written to calculate thermodynamic data for reactions within an SBML model, called the Thermodynamic Analysis Tool (ThAT). For each reaction in the given metabolic model, a standard Gibbs free energy of reaction (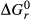) as well as a temperature-dependant Δ*G*_*r*_ was calculated for either 303.15K (30 °C) or 278.15K (5 °C). To calculate Δ*G*_*r*_, two data sets were used, the first was a list of Δ*G*_*f*_ from metabolites found in *Escherichia coli,* from the supplementary material of Jankowski *et al*. (Jankowski *et al*. 2008). However, not all metabolites have an associated Δ*G*_*f*_ as some of them have generic radical groups and other side chains that are not uniquely characterized. The other list was a range of metabolite concentrations for those metabolites with measured values (Albe *et al*. 1990). Where no data were available an estimate of upper and lower metabolite concentration was used by selecting the second lowest and second highest metabolite concentrations from Albe *et al*. which gave values that reflected realistic biological conditions while taking into account possible outliers. The low and high concentration bounds chosen were 0.00001 and 0.02 m. The Δ*G*_*f*_ data were in k cal/mol, so a value of 1.9858775 k cal/mol was used for the gas constant (R); temperatures were expressed in Kelvin.

In this study, the *Saccharomyces cerevisiae* metabolic reconstruction model *i*MM904 was used (Mo *et al*. 2009). This model is compartmentalized and covers 904 genes and 1412 reactions. Approximately 22% of the reactions in the model could not have their Δ*G*_*r*_ calculated as they contained one or more metabolite that lacks an associated Δ*G*_*f*_ value; these reactions were classified as ‘undetermined’ for the thermodynamic analysis.

### Strains used in this study

*Saccharomyces cerevisiae* heterozygous yeast deletion collection based on BY4743 (*MAT*a/α *his3*Δ*1/his3*Δ*1 leu2*Δ*0/leu2*Δ*0 LYS2/lys2*Δ*0 met15*Δ*0/MET15 ura3*Δ*0/ura3*Δ*0*), *S. cerevisiae* 96.2 natural isolate and *S. kudriavzevii* natural isolate. Both these isolated from *Quercus ilex* bark in Castellón, Spain (courtesy of E. Barrio).

### Genome profiling

A set of continuous culture experiments were carried out as described by (Delneri 2011). Small volume parallel fermentation vessels were used as chemostat system (DASGIP technology, an Eppendorf company, Jülich, Germany). Three media were used: rich medium (YPD), F1 Nitrogen-limited medium and F1 carbon-limited medium, as described in Table S1 (Supporting information) (Delneri 2011). The medium was pumped into an autoclaved 20 l barrel through a Sartopore 2 150 filter, 0.2 μm (Sartorius). A 0.5 l sample of the medium was pumped into clean autoclaved bottles for testing for contamination (5 ml of medium was put in a universal tube and incubated overnight at 30 °C) and for filling the chemostat vessels (120 ml each).

The pool of heterozygous BY4743 diploid *S. cerevisiae* mutants containing ∼300 cells per deletant strain was used to inoculate the chemostat's vessels. The vessels were kept in an incubator at 16 °C. The culture was grown in batch for up to 3 days (2 days for rich YPD medium and 3 days for the two F1 limited medium) before switching to continuous conditions. A pump was used to introduce fresh media into the vessels at a constant rate with a dilution rate 0.05/h (∼2 generations a day). Another line was used to pump out any surplus media, and it was mechanically regulated by the volume of the culture which remains constant throughout the experiment. A third pump fed 1 m KHO into the vessels to maintain the pH at the set value of 4.5 and was computationally controlled.

The experiment was carried out for 2 weeks after switching to continuous culture so that final sample contained a population of cells grown for ∼28 generations after the batch phase. A sample of the initial culture (used to inoculate all the flasks) was kept, and then for each experiment, samples were taken at the batch phase, at the beginning of the steady-state phase and then every other day until the last day when all remaining culture was collected. Samples from the vessels were taken via the waste tubes so that the culture volume was kept constant and the steady state was not disturbed.

The number of strains present in the population, and therefore the effect that each mutation have on the organism fitness, was evaluated via the ‘Bar-seq’ method using SOLiD platform and bioinformatics analysis (Smith *et al*. 2009). DNA was extracted from samples using the Promega Wizard DNA extraction kit, and for each condition (rich medium, carbon-limited medium and nitrogen-limited medium), two biological replicates were extracted. The upstream tags were amplified using the SOLiD primers and prepared for SOLiD sequencing, as described by Smith *et al*. (Smith *et al*. 2010). The PCR products were cleaned up using the Qiagen PCR clean up kit and then quantified using a fluorescence assay designed to detect double stranded DNA (Quant-iT™ PicoGreen® dsDNA Reagent and Kit, Invitrogen). Each library was diluted to 10 μg/ml and equal volumes of each pooled together. The pool was run on a 10% TBE polyacrylamide gel (Invitrogen, Paisley, UK) to check the length of the PCR products. The sequencing results were then tabulated normalized and compared with the original pool values, for further details, see Data S1 (Data analysis of competition experiment, Supporting information).

### Creation of sympatric mutants

A sympatric pair of natural isolate yeasts, *S. cerevisiae 96.2* and *S. kudriavzevii* CA111, were used for temperature fitness assays (courtesy of Eladio Barrio). Heterozygote mutants were created separately in the two yeast backgrounds according to Gietz *et al*. (Daniel Gietz & Woods 2002). The knockout was achieved by inserting a PCR-created *kanMX* cassette into the yeast genome (Wach *et al*. 1994), and the oligonucleotides used for the deletions are listed in the Table S2 (Supporting information). For *S. kudriavzevii* CA111, the heat shock was carried out at 37 °C rather than 42 °C, and the incubation was carried out at 27 °C. To create homozygote mutants, the heterozygote deletant strains were sporulated for either 2–3 days (*S. cerevisiae* 96.2) or 7–10 days (*S. kudriavzevii* CA111). The tetrads were then dissected using a micromanipulator and left to self-fertilize to create the homozygote strains, which were tested by PCR to check that both copies of the gene of interest were removed (the designed checking oligonucleotides are listed in Table S2, Supporting information).

### Creation of overexpression mutants

*ADH3* and *GUT2* were TA cloned into pCR™2.1 Vector (Invitrogen) using primers detailed in Table S2 (Supporting information). The vectors were transformed into One Shot® INVαF’ Chemically Competent *E. coli* (Invitrogen). The genes were then cloned into pRS315 (*GUT2*) and pRS316 (*ADH3*) backbones containing the *TDH3* constitutive promoter using suitable restriction enzymes (a list of plasmids used in this work can be found in Table S3, Supporting information). These plasmids along with the empty vectors were transformed individually into BY4743.

### Temperature assay experiments

For the fitness studies, the strains of interest were grown overnight and then transferred to a 96-well microplate with an initial OD_595_ of 0.1. A FluroStarOptima bioscreen machine (BMG Labtech, Offenberg, Germany) was used to score the growth curves at OD_595_. The experiments were carried out at both 12 and 30 °C in the chemical defined limited media F1, Table S1 (Supporting information) (Delneri 2011). For the colder temperatures, the bioscreen was kept in a cooling incubator set at 12 °C. *S. kudriavzevii* fitness assays at 12 and 30 °C and *S. cerevisiae* fitness assays at 12 °C were carried out over 72 h while the *S. cerevisiae* fitness assay at 30 °C was carried out over 48 h, since it reached stationary phase earlier. For all experiments, three biological (different transformants colonies for the same mutation) and three technical replicates (different cultures of the same transformant) were used, and the error bars for these growth curves are the standard deviation of the replicates from the mean growth curve.

### Measurement of genetic expression

Real-time PCR was used to quantify gene expression of *AHD3* and *GUT2* in both *S. cerevisiae 96.2* and *S. kudriavzevii* CA111 at both 30 and 12 °C. Each species was grown to mid-log-phase and the total RNA extracted using RNeasy kit (Qiagen, Hilden, Germany). Real-time PCR was conducted using iScript™ One-Step RT-PCR Kit with SYBR® Green from Biorad and analysed on a Biorad CFX Connext Real-Time System (Biorad, Hemel Hempstead, UK). Expression levels were normalized to *ACT1* values corresponding to the relevant species and temperature to obtain relative expression data.

### Glycerol assay

To quantify extracellular glycerol, *S. kudriavzevii* CA111 wild type and *S. kudriavzevii* CA111 *ΔYIL155c/ΔYIL155c* were grown in F1 media at 12 °C until the stationary phase was reached. Samples of the media were filter sterilized to remove the yeast cells and a UV-assay kit was used to measure glycerol levels (R-Biopharm, Darmstadt, Germany).

## Results

### Whole-genome analysis of *S. cerevisiae* thermodynamics

#### Thermodynamics Analysis Tool (ThAT)

An initial investigation into temperature-dependant Δ*G* was conducted to compare the Gibbs–Helmholtz equation (Equation 1) and the quotient rule equation (Equation 2). The Gibbs–Helmholtz equation relies on the availability of measured Δ*H* values; however, only 3% of the 877 metabolites in the *i*MM904 genome-scale model have a trusted Δ*G* value (Alberty 2003). Thus, only a very limited number of reactions could have their temperature-dependant Δ*H* calculated. This is not suitable for a comprehensive coverage of the genome-scale network of yeast. 
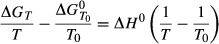
1

where Δ*G*_*T*_ is the Gibbs energy at the new temperature, 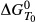 is the standard Gibbs free energy at 297.15K, *T* is new temperature, *T*_0_ is the standard temperature and Δ*H*^0^ is the standard enthalpy. 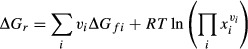
2

where *v*_*i*_ is the stoichiometric coefficient of metabolite *i*, Δ*G*_*fi*_ is the Gibbs free energy of formation of metabolite *i*, *R* is the gas constant, *T* is the temperature and *x*_*i*_ the concentration of metabolite *i*.

The quotient rule is dependent on temperature and metabolite concentrations where again, there is a limited amount of experimental data to inform this equation. Using the BioNumbers database (Milo *et al*. 2010), it was possible to assign metabolite concentration ranges for 4% of the 877 metabolites in the model (Albe *et al*. 1990). Nevertheless, an estimate of the range of concentrations can be used for the remaining metabolites (Henry *et al*. 2006).

We investigated varying the concentrations of individual components of each reaction and calculated Δ*G*_*r*_ values for all possible combinations of metabolite concentrations. However, we hypothesized that it would be biologically unfavourable to have large gaps between concentrations of metabolites participating to the same reaction, with the exception of ubiquitous metabolites such as ATP, and that such conditions would be unlikely at steady states in vivo. Thus, to calculate a range of possible Δ*G*_*r*_ values for a reaction, the metabolite concentrations were fixed at eit-her the high or the low concentration level for all metabolites.

Most of the Δ*G*_*r*_ calculated using the Gibbs–Helmholtz equation lie within or close to the range calculated using metabolite activity bounds at both temperatures tested (Tables S4 and S5, Supporting information). For this study, it was important to calculate Δ*G*_*r*_ for as many reactions within the *S. cerevisiae* metabolite network as possible. Thus, the quotient equation (Equation 2) was used as the data needed to calculate Δ*G*_*r*_ has a wider coverage, and the range of Δ*G*_*r*_ covered by considering lower/upper concentration bounds was found to encompass variations due to the enthalpy. The analysis tool designed to analyse thermodynamics of a metabolic model in this fashion was named Thermodynamic Analysis Tool (ThAT).

#### Application of ThAT to metabolic reactions

The Δ*G*_*r*_values calculated at 5 °C were compared with values calculated at 30 °C (Table S6, Supporting information) for all 1,412 reactions in the *Saccharomyces cerevisiae* metabolic network. In principle, all reactions are reversible, and a change in temperature may cause a change of reaction direction, thus directionality of a reaction was not considered when assigning temperature favourability. A cold-favoring reaction was defined as a reaction whose products or substrates are more energetically favoured at a colder temperature than at a warmer one. Using the Δ*G*_*r*_ data, a reaction was deemed cold favouring if the absolute value of Δ*G*_*r*_ at 5 °C minus the absolute value of Δ*G*_*r*_ at 30 °C was >0 (Table S5, Supporting information). This means that when both values of Δ*G* are positive, it is the backward reaction that is cold favouring since the substrates, rather than the products, are more energetically favourable. This definition is used as a qualitative measure to classify reactions as cold favouring, warm favouring or undecided; we did not used it to quantify how the gene may respond to temperature, as there is no direct quantitative relation between Δ*G*_*r*_ and reaction rate.

As the ThAT calculates an upper and lower bound for the Δ*G*_*r*_ value it was necessary to consider both when determining temperature preference. Each reaction was analysed for cold favouritism at high and low metabolite concentration. Only reactions that preferred cold at both metabolite conditions were considered fully cold favouring. By only considering reactions as cold favouring, if they meet the requirements in both high and low concentration, the classification is rigorous and eliminates cases where the difference due to temperature change is too small.

From the *S. cerevisiae i*MM904 model, 46 reactions were classified as cold favouring, a list detailing these reactions can be found in Table1. We compared the list of predicted cold-favoring genes to published genomic expression data of *S. cerevisiae* under heat shock at 37 °C (Gasch *et al*. 2000). The expression of each gene was measured by Gasch *et al*. at 5, 10, 15, 20, 30, 40 and 60 min after the cells were shocked using microarrays.

**Table 1:** List of cold-favouring reactions predicted by the thermodynamic analysis

Reactions	ORFs	Gene names	Pathways	EC numbers
Adenylosuccinate lyase: 2-[5-amino-1-(5-phospho-D-ribosyl)imidazole-4-carboxamido]succinate -> fumarate	*YLR359w*	*ADE13*	Purine and pyrimidine biosynthesis	4.3.2.2
Adenylosuccinate lyase: 1-2-Dicarboxyethyl.AMP ->fummate				
Alcohol dehydrogenase: forward rxn: ethanol->acetaldehyde	*YMR303c*	*ADH2*	Pyruvate metabolism	1.1.1.1
Alcohol dehydrogenase: reverse rxn: acetaldehyde->ethanol	*YOL086c YGL256w YBR145w*	*ADH1 ADH4 ADH5*	Pyruvate metabolism	1.1.1.1
Alcohol dehydrogenase: reverse rxn: acetaldehyde->ethanol	*YMR083w*	*ADH3*	Pyruvate metabolism	1.1.1.1
Alcohol dehydrogenase: ethanol	*YDL168w*	*SFA1*	Pyruvate metabolism	1.1.1.1
Adenosine monophosphate deaminase	*YML035c*	*AMD1*	Purine and pyrimidine biosynthesis	
Biotin acetyl-CoA carboxylase ligase	*YDL141w*	*BPL1*	Pantothenate and CoA biosynthesis	6.3.4.15
Chorismate synthase	*YGL148w*	*ARO2*	Tyrosine, tryptophan and phenylalanine metabolism	4.2.3.5
Chorismate pyruvate lyase	Unspecified in model	Quinone b	Unspecified	
Cystathionine b lyase	*YFR055w*	*CYS1*	Methionine metabolism	4.4.1.8
Cystathionine b lyase: peroxisomal	*YGL184w*	*STR3*	Methionine metabolism	4.4.1.8
3 dehydroquinate synthase	*YDR127w*	*ARO1*	Tyrosine tryptophan and phenylalanine metabolism	4.2.3.4
Fatty acid: CoA ligase: tetradecanoate	*YOR317w YIL009w YMR246w*	*FAA1 FAA3 FAA4*	Fatty acid biosynthesis	6.2.1.3
Fatty acid: CoA ligase: tetradecanoate: peroxisomal	*YER015w*	*FAA2*	Fatty acid biosynthesis	6.2.1.3
Fructose bisphosphate aldolase	*YKL060c*	*FBA1*	Glycolysis and gluconeogenesis	4.1.2.13
D Fructose 1 phosphate D glyceraldehyde 3 phosphate lyase				
Sedoheptulose 1,7-bisphosphate D glyceraldehyde 3-phosphate lyase				
FMN adenylyltransferase	*YDL045c*	*FAD1*	Riboflavin metabolism	2.7.7.2
FMN adenylyltransferase: mitochondrial				
Glycerol 3 phosphate dehydrogenase: FAD: mitochondrial	*YIL155c*	*GUT2*	Glycerolipid metabolism	1.1.99.5
Glycogen phosphorylase	*YPR160w*	*GPH1*	Alternate carbon metabolism	2.4.1.1
Glutamate dehydrogenase: NAD	*YDL215c*	*GDH2*	Glutamate metabolism	1.4.1.2
GTP diphosphohydrolase	*YER005w*	*YND1*	Nucleotide salvage pathway	3.6.1.5
IMP dehydrogenase	*YHR216w YLR432w YAR075w YML056c YAR073w*	*IMD2 IMD3 IMD5 IMD4 IMD1*	Purine and pyrimidine biosynthesis	1.1.1.205
Malic enzyme: NAD: mitochondrial	*YKL029c*	*MAE1*	Anaplerotic reactions	1.1.1.38
Sulphate adenylyltransferase	*YJR010w*	*MET3*	Cysteine metabolism	2.7.7.4
Methylenetetrahydrofolate dehydrogenase: NAD	*YKR080w*	*MTD1*	Folate metabolism	1.5.1.5
Cytochrome P450 lanosterol 14 alpha demethylase: NAD	*YNL111c YHR007c YKL150w YIL043c YNL111c YHR007c*	*CYB5 ERG11 MCR1 CBR1 CYB5 ERG11*	Sterol metabolism	1.14.14.1
Methylenetetrahydrofolate dehydrogenase: NAD	*YKR080w*	*MTD1*	Folate metabolism	1.5.1.5
Nucleoside diphosphatase: GDP	*YER005w*	*YND1*	Nucleotide salvage pathway	3.6.1.6
Nucleoside diphosphatase: UDP				
UTP diphosphohydrolase				
Nucleoside diphosphatase: GDP: Golgi	*YEL042w*	*GDA1*	Nucleotide salvage pathway	3.6.1.6
Nucleoside diphosphatase: dGDP				
Nicotinate nucleotide adenylyltransferase	*YLR328w*	*NMA1*	NAD biosynthesis	2.7.7.18
Nicotinate nucleotide adenylyltransferase: mitochondrial				
Nucleoside triphosphatase: GTP	Unspecified in the model	Nucleotide salvage pathway	3.6.1.15	
Nucleoside triphosphatase: dGTP		Nucleotide salvage pathway	3.6.1.15
Phosphor-ribosyl-formyl-glycinamidine synthase	*YGR061c*	*ADE6*	Purine and pyrimidine biosynthesis	6.3.5.3
Pantetheine phosphate adenylyltransferase	Unspecified in the model	Pantothenate and CoA biosynthesis	2.7.7.3	
Pantetheine phosphate adenylyltransferase		Pantothenate and CoA biosynthesis	2.7.7.3
D1 pyrroline 5 carboxylate dehydrogenase: mitochondrial	*YHR037w*	*PUT2*	Glutamate metabolism	1.5.1.12
L serine deaminase	*YCL064c YIL168w*	*CHA1 SDL1*	Glycine and serine metabolism	4.3.1.17
L allo Threonine Aldolase	*YEL046c*	*GLY1*	Threonine and lysine metabolism	4.1.2.5
Threonine aldolase				

A χ^2^ test for differences was used to compare the percentage of genes that were up (a fold change of over 0.5) or down (a fold change of less than −0.5) regulated in the heat-shock data set by Gasch *et al*. with our list of cold-favoring genes predicted by ThAT. Our cold-favoring genes were significantly under-represented among the upregulated genes (*P*-value <0.05) and significantly overrepresented among the downregulated genes (*P*-value <0.001) in the heat-shock data set (Gasch *et al*. 2000). Our warm favouring gene list showed the opposite trend. These values support the hypothesis that the cold predicted genes would be downregulated in heat shock and the warm predicted genes would be upregulated.

Based on the thermodynamic prediction, a Gene Ontology (for details see Data S1, Supporting information) analysis of potential cold-favouring genes was carried out using the online tool DAVID (da Huang *et al*. 2009a,b) using the *i*MM904 gene list as the background. This tool identified 16 significantly (*P*-value <0.05) enriched functional categories, shown in Table2. NAD-related genes were the most significant category with a *P*-value of 2.58E-09. The next most enriched functional categories were alcohol metabolism, purine biosynthesis and oxidoreductase. The list also included lipid metabolism and fatty acid metabolism that are essential for membrane fluidity, a well-known factor involved in temperature acclimation.

**Table 2:** Gene Ontology analysis of the cold-favouring genes predicted by the thermodynamic analysis

Functional categories	*P*-value
NAD	2.58E-09
Alcohol Metabolism	1.34E-05
Purine Biosynthesis	8.23E-05
Oxidoreductase	1.40E-04
CBS Domain	7.19E-04
GMP Biosynthesis	7.19E-04
Metalloprotein	8.53E-04
Potassium	5.35E-03
Metal-Binding	6.55E-03
Zinc	7.54E-03
Purine Nucleotide Biosynthesis	9.88E-03
Fatty Acid Metabolism	1.28E-02
Lipid Metabolism	2.00E-02
Lyase	4.30E-02

### Genome profiling of the heterozygote yeast deletion collection at cold temperature

Continuous culture experiments using the BY4743 diploid heterozygote *S. cerevisiae* mutant collection were carried out in three media types at 16 °C to identify genes that affect competitive growth at cold temperature. If a gene is a key player in controlling the flux through a specific pathway, it is expected that lowering its dosage from 2 to 1 will impair the cell growth compared with other genes which have low control (Delneri *et al*. 2008). Therefore, the competition data can be used to identify genes involved in regulating the metabolic flux, revealing pathways that are important for growth at cold temperature.

**Figure 2:**
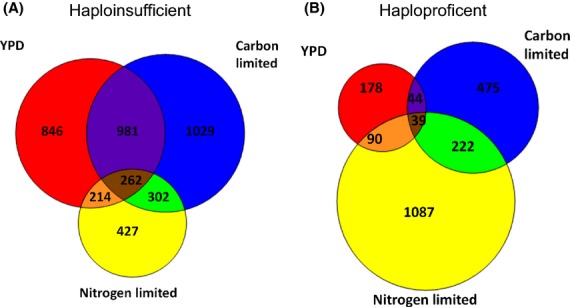
Venn diagrams to show the overlap of the unique genes that were haplo insufficient (A) or haploproficient (B) in the 16 °C genome profiling in rich media (YPD), carbon limited of nitrogen limited.

**Figure 3:**
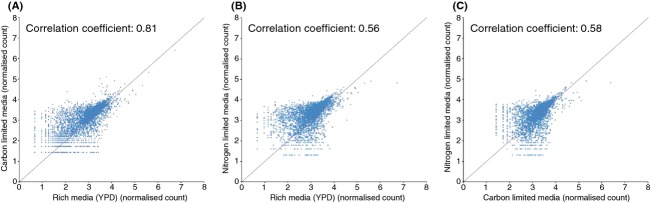
Scatter plots barcode count data from final steady-state samples. A comparison of rich media with carbon-limited media (A) or nitrogen-limited media (B) as well as a comparison of the two limited conditions (C). The panels also include the correlation coefficient of the corresponding data sets.

Overall, 265 genes were found to be haploinsufficient (at least 1.5-fold decrease in copy number) in all three media conditions but only 39 were haploproficient (at least 1.5-fold increase in copy number). The number of uniquely haplo insufficient and haploproficient genes is summarized in Fig.2, and the full list of fold-change values are listed in Table S8 (Supporting information). In rich media, approximately 6% of genes were haploproficient, whereas in carbon-limited and nitrogen-limited conditions ∼13 and ∼24% were haploproficient, respectively. There was a larger overlap of haploinsufficient genes between carbon-limited and YPD condition compared with the other media. Overall, in nitrogen-limited conditions, there were half of the haploinsufficient genes detected compared with the other condition. This large difference between the nitrogen-limited conditions and the other media types is also shown in the correlations between overall numbers of barcode counts (Fig.3). The rich media and carbon-limited conditions showed the highest correlation coefficient of 0.81 (Fig.3A), which is comparable with the variation between biological replicates that have an average correlation coefficient of 0.8 (example plots are shown in Fig. S1, Supporting information). The nitrogen-limited data showed less similarity with rich media and carbon-limited media, with a correlation coefficient of 0.56 and 0.58, respectively (Fig.3B,C).

Gene ontology (GO) analysis (for details, see Data S1, Supporting information) was carried out on the list of genes whose mutants were significantly affected by the cold temperature in all three media conditions (Table3). The analysis showed that lipid biosynthesis genes were significantly enriched (*P*-value <0.05). Oxidoreductase-related reactions were also found to be significantly enriched, and this was consistent with the GO analysis of the gene list predicted by the thermodynamic analysis.

**Table 3:** Gene Ontology of mutants affected by cold temperature identified by the genome profiling experiment

Functional categories	*P* value
Isopeptide Bond	7.86e-04
UBL Conjugation	5.43e-03
Transport	5.63e-03
Oxidoreductase	5.81e-03
Protein Phosphatase	8.83e-03
Membrane Protein	1.03e-02
Nucleus	1.28e-02
Zinc Finger	2.00e-02
Riboflavin Biosynthesis	2.14e-02
DNA Repair	2.55e-02
FMN	2.62e-02
Iron	2.66e-02
DNA Binding	2.75e-02
NAD	3.27e-02
Protein Transport	3.27e-02
Monooxygenase	3.44e-02
Chromosomal Protein	3.57e-02
Nucleotide Binding	3.63e-02
Homodimer	3.64e-02
mRNA Transport	3.81e-02
P-Loop	4.03e-02

We found that vitamin metabolism was overrepresented among the genes affected by cold temperature. For example, riboflavin (vitamin B2) biosynthesis was one of the vitamin pathways that contained a significant number of genes that affected fitness in cold conditions. As vitamins are coregulators for a wide range of reactions, this may indicate that more processes are important in cryo-tolerant species.

### Growth assay of *Saccharomyces cerevisiae* 96.2 and *Saccharomyces kudriavzevii* CA111 *ADH3* and *GUT2* mutants

The list of cold-favouring reactions from the thermodynamic analysis was combined with the fold-change data from the competition experiment to identify genes that were significantly haploinsufficient in two or more media conditions and were never haploproficient in any condition (Table4). The genes that had the largest control over the metabolic flux in the three media types at 16 °C were *GUT2* (*YIL155c*) and *ADH3* (*YMR083w*). Both genes have orthologs in *S. kudriavzevii***,** and we expected that mutation in any of them would have a larger deleterious effect in this cold-tolerant species compared with *S. cerevisiae* 96.2. Heterozygote and homozygote gene knockout strains of *GUT2* and *ADH3* were created in the natural isolate *S. cerevisiae* 96.2 and *S. kudriavzevii* CA111, and the phenotypic fitness assays were performed using a microplate reader scoring the yeasts growth both at 30 and 12 °C. The fitness of the mutants was quantified by calculating the area under the growth curve (Norris *et al*. 2013) and compared with the corresponding value of the parental strain (Table5).

**Table 4:** List of genes displaying the highest fitness impairment at 16 °C after combining both thermodynamic and genome screening data

ORFs	Fold change of cell growth (log_2_)		
	YPD	Carbon	Nitrogen
*ADH3*	−1.82628	−0.56671	−0.08505
*GUT2*	*	−1.09477	−0.95155
*NMA*	−0.91595	−0.96021	0.184467
*YND1*	−0.86889	−0.56397	0.374916
*ADH5*	−0.80525	−0.57228	−0.05321
*SFA1*	−0.53395	−0.66071	−0.00589
*GPH1*	−0.05416	−0.7147	−0.62748

* Data not available due to technical issues.

**Table 5:** List of ratios of area under the growth curve between the *ADH3* and *GUT2* mutants compared with their respective wild types

Strain	Ratio to wild-type parent at 12 °C	Ratio to wild type at 30 °C
*S. cerevisiae 96.2*	1.00	1.00
*S. cerevisaie ADH3/ΔADH3*	0.73	0.99
*S. cerevisaie ΔADH3/ΔADH3*	0.64	1.02
*S. cerevisaie GUT2/ΔGUT2*	1.01	1.08
*S. cerevisaie Δ GUT2/ΔGUT2*	0.87	1.05
*S. kudriavzevii CA111*	1.00	1.00
*S. kudriavzevii CA111 ADH3/ΔADH3*	0.72	2.19
*S. kudriavzevii CA111 ΔADH3/ΔADH3*	0.71	1.75
*S. kudriavzevii CA111 GUT2/ΔGUT2*	0.91	2.73
*S. kudriavzevii CA111 ΔGUT2/ΔGUT2*	0.57	1.71

**Figure 4:**
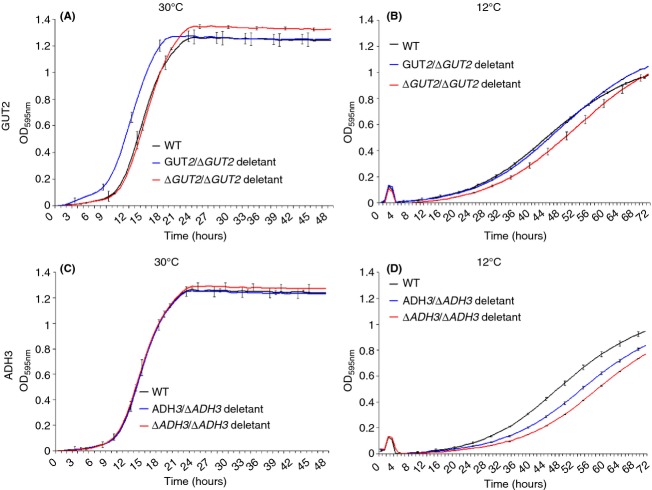
Growth curves of *S. cerevisiae* 96.2 mutants in F1 media at different temperatures. *GUT2* mutants grown at 30 °C (A) and 12 °C (B) and *ADH3* mutants grown at 30 °C (C) and 12 °C (D) are shown. For each mutant, three biological and three technical replicas were analysed. 49 independent data points were plotted for mutants grown at 30 °C (Panel A and C), while 73 data points were plotted for mutants grown at 12 °C (Panel B and D). The error bars represent the standard deviation from the average.

At 30 °C, when compared to the wild type, *S. cerevisiae* 96.2 *GUT2* heterozygote and homozygote mutants showed a small advantage in the lag phase and in the final biomass, respectively, (Fig.4, Panel A). When considering the overall fitness (i.e. lag phase shift, growth rate and final biomass), the difference between mutants and wild type was minimal (Table5). At 12 °C, only the *S. cerevisiae* 96.2 *GUT2* homozygote mutant showed a drop in overall growth (Fig.4, Panel B), while both heterozygote and homozygote *ADH3* mutants had a greater fitness loss than *GUT2* mutants (Table5 and Fig.4, Panel D).

**Figure 5:**
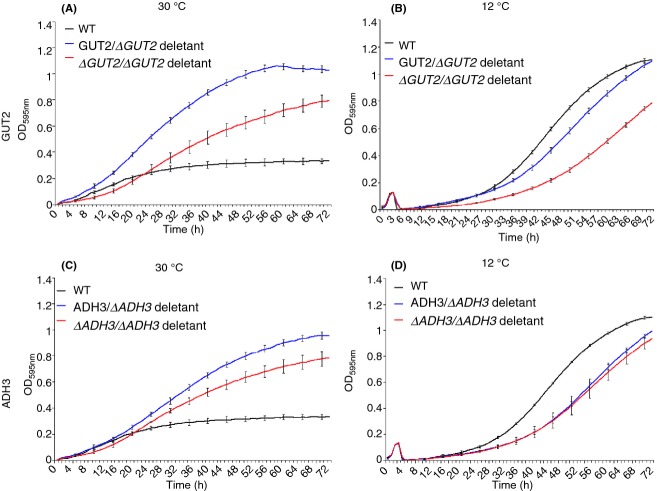
Growth curves of *Saccharomyces kudriavzevii* CA111 mutants in F1 media at different temperatures. *GUT2* mutants grown at 30 °C (A) and 12 °C (B) and *ADH3* mutants grown at 30 °C (C) and 12 °C (D) are shown. For each mutant, three biological and three technical replicas were analysed, and 73 data points were plotted. The error bars represent the standard deviation from the average.

All mutant strains created in *S. kudriavzevii* CA111 background (heterozygotes and homozygotes mutants for both *ADH3* and *GUT2*) showed a drop in fitness at 12 °C (Fig.5, Panel B and D, Table5); however, the *GUT2* deletion strains had a much greater growth disadvantage compared with the *ADH3* mutants. Both heterozygote and homozygote *ADH3* deletion strains grew at about the same rate, showing a comparable fitness loss (Fig.5).

Strikingly, at 30 °C, all the *S. kudriavzevii* CA111 mutants grew remarkably better than the wild-type parent (Fig.5, panel A and C). The *ADH3* and *GUT2* homozygote mutants had a smaller fitness advantage compared with the herterozygotes, suggesting that the complete removal of the gene may interfere with other biological functions in the cell.

Overall, these data not only confirm that, by removing genes associated with predicted cold-favoring reactions, yeast fitness is decreased at low temperature. Moreover, deletion of *ADH3* and *GUT2* conferred resistance to *S. kudriavzevii* CA111 at higher temperatures (Figs4 and 5). By knocking down the cold-favouring genes, this species increased its ability to grow at 30 °C reaching a fitness comparable to its thermo-tolerant closely related species.

### Expression of *ADH3* and *GUT2* genes in *Saccharomyces* natural isolates

**Figure 6:**
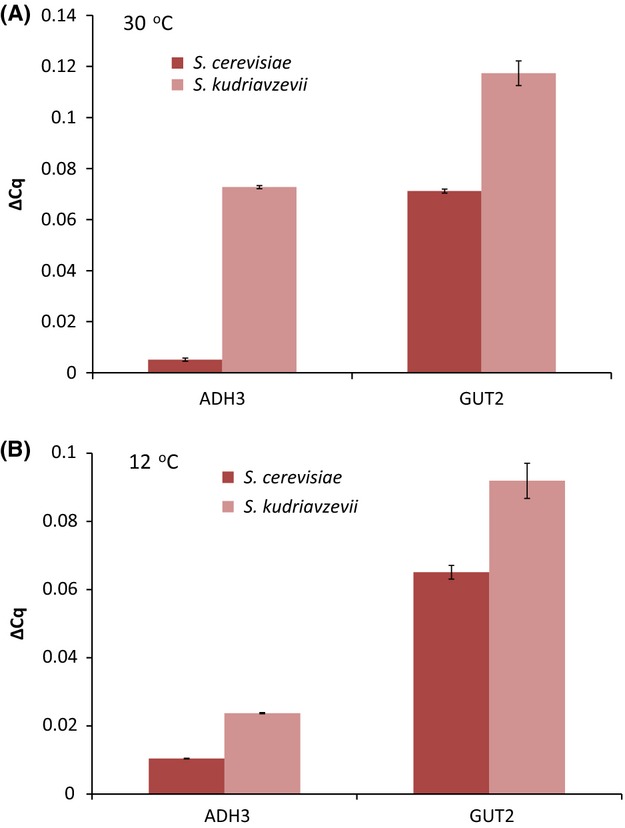
Relative expression of *GUT2* and *ADH3* in *Saccharomyces cerevisiae* 96.2 and *S. kudriavzevii* CA111 at 30 °C (A) and 12 °C (B). For each gene, two biological and three technical replicas were analysed. The error bars represent the standard deviation from the average.

The genome profiling study identified genes whose dosage is important for maintaining the cold phenotype. It is therefore possible that different levels of expression of *ADH3* and *GUT2* in *S. kudriavzevii* CA111 and *S. cerevisiae 96.2* are partially responsible for cryo-tolerance. We would expect that both *ADH3* and *GUT2* are expressed at higher level in the cold-tolerant species, and we quantitatively measured the level of mRNA for these two genes via real-time PCR in both natural isolates grown at 30 and 12 °C. We found that *ADH3* had extremely low expression at 30 °C in *S. cerevisiae* 96.2, due to glucose repression (Young & Pilgrim 1985), while in *S. kudriavzevii* CA111 showed a 14-fold increase in expression at 30 °C compared with *S. cerevisiae* 96.2 (Fig.6A). Interestingly, these data suggest that an efficient glucose repression mechanism for *ADH3* is not present in *S. kudriavzevii* CA111. At 12 °C, a similar profile was seen for *ADH3,* which was more expressed in *S. kudriavzevii* CA111 than in *S. cerevisiae* 96.2. *GUT2* expression was also higher in *S. kudriavzevii* CA111 at both temperatures, although to a lesser extent than *ADH3* (Fig.6). To investigate whether overexpression of *ADH3* and *GUT2* could increase growth at cold, we cloned *ADH3* and *GUT2* genes into pRS315 and pRS316 backbone plasmids containing the strong constitutive promoter, *TDH3* (detailed in Table S3, Supporting information). The overexpression vectors were transformed into the wild-type *S. cerevisiae* BY4743 strain, and fitness profiles at 30 and 12 °C were scored.

At 30 °C, the strain overexpressing *ADH3* showed no significant phenotypic change; however, at 12 °C, the overall fitness increased by 15% (Table6 and Fig. S2, Supporting information). This result indicates that while overexpression of *ADH3* does not confer any advantage at 30 °C, it does have a positive effect on the overall growth at cold. The *GUT2* overexpression strains was not significantly different than the wild type (less than one standard deviation difference), at either temperature (Table6 and Fig. S2, Supporting information). These expression-based data show that both *GUT2* and *ADH3* are more highly expressed in *S. kudriavzevii* CA111 when compared to *S. cerevisiae 96.2* and that *ADH3* is not efficiently repressed by glucose in *S. kudriavzevii*. Moreover, overexpression of *ADH3* in *S. cerevisiae* increases its cryo-tolerance.

**Table 6:** List of ratios of area under the growth curve for overexpression mutants of *ADH3* and *GUT2* measured at 30 and 12 °C compared with their wild-type parental strains. ^*^not significant due to noisy data

Strain	Ratio to wild-type *S. cerevisiae* at 12 °C	Ratio to wild-type *S. cerevisiae* at 30 °C
BY4743 ADH control	1.00	1.00
BY4743 + *ADH3*	1.15	0.99
BY4743 GUT2 control	1.00	1.00
BY4743 + *GUT2*	0.88^*^	1.00

## Discussion

In this study, a novel approach was undertaken to identify genes that alter the thermoprofiles of *Saccharomyces cerevisiae* and *Saccharomyces kudriavzevii*. By integrating thermodynamic modelling with a metabolic yeast reconstruction, 46 potential cold-favouring reactions were identified. Previous work has used thermodynamic analysis to identify reactions that are energetically unfeasible at a given temperature (Henry *et al*. 2006; Jol *et al*. 2012), and others have used various temperatures on a specific pathway (Cruz *et al*. 2012), but none have considered multiple temperatures on the genome scale. The list of potential cold-favoring reactions contained a number of genes that were related to mitochondrial, fatty acid and lipid metabolisms, all of which have previously been documented to be important pathways for temperature adaptation in other organisms (Vigh *et al*. 1998; Los & Murata 2004; Schade *et al*. 2004). In addition, using ThAT, we identified oxidoreductase reactions as important for growth at cold temperature.

Comparison studies between our list of predicted genes with available heat-shock transcriptome data (Gasch *et al*. 2000) showed that a significant number of cold-favouring genes were downregulated during heat shock, while genes predicted to be warm favouring by ThAT were significantly upregulated in heat shock. These results suggest that our thermodynamic analysis can identify genes important for regulation of cell growth at nonoptimal temperature.

Gene ontology (GO) analysis of haploinsufficient and haploproficient genes, identified through the large-scale competition experiment at 16 °C, revealed an enrichment of pathways similar to those identified by the thermodynamic analysis, in particular lipid biosynthesis- and oxidoreductase-related reactions.

In addition, vitamin metabolism was also a highly enriched GO category. For example, the riboflavin biosynthetic pathway was clearly affected by the cold conditions. Riboflavin is used to synthesize flavin mononucleotide (FMN) and flavin adenine dinucleotide (FAD) which are cofactors that are readily oxidized or reduced for enzymatic purposes (Ghisla & Massey 1989). A disruption to the riboflavin pathway may cause a change in the production of FAD and FMN possibly leading to a redox imbalance, which in turn causes decreased fitness. According to the Kyoto Encyclopaedia of Genes and Genomes database (KEGG) (Kanehisa 2000), there are nine riboflavin genes that are present in *S. cerevisiae*, seven of which were found haploinsufficient in our genomic screen in YPD (*YBL033C, YDR487C, YOL143C, YBR153W, YOL066C, YBR256C* and *YDL045C*). Vitamins may have a role in growth at cold temperatures through their regulation of fatty acids. For example, vitamin H (biotin), essential for *S. cerevisiae* growth, is a cofactor for the carboxylase family, and the acetyl-coA carboxylase gene (*ACC1)* is a biotin mediated step at the beginning of the fatty acid biosynthesis pathway. Interestingly, in our genomic screen at 16 °C, the *ACC1* mutant had a striking decrease in fitness in both YPD and carbon-limited medium (no change detected in nitrogen-limited condition), while in previous competition experiments at 30 °C, the *ACC1* mutant showed no fitness defect in carbon-limited medium and a slightly but significant gain of fitness in the nitrogen-limited condition (Delneri *et al*. 2008). Taken together, these results indicate that the biotin mediated step is crucial for the yeast fitness at lower temperatures.

*Saccharomyces kudriavzevii* is a closely related species of *S. cerevisiae* which evolved an optimal growth temperature 8 °C lower than that of *S. cerevisiae*, thus it may favour reactions and pathways whose products are more stable or more energetically favourable at colder temperatures. This implies that genes coding for enzymes catalysing these reactions have become more important to this yeast's survival than those that have a similar function in *S. cerevisiae*. Two potential cold adaptation genes, *ADH3* and *GUT2*, were identified from genome-scale analyses and hetero zygous and homozygous mutants for these genes were created in the two natural yeast isolates, *S. cerevisiae* 96.2 and *S. kudriavzevii* CA111. *ADH3* mutants had a similar effect on the fitness in both *S. cerevisiae* and *S. kudriavzevii* background at 12 °C showing a decrease in fitness compared with respective wild natural isolates. The *GUT2* mutants in a *S. cerevisiae* 96.2 background displayed little fitness defect; however, in the *S. kudriavzevii* background, there was a significant impairment of fitness at 12 °C. In addition to this, overexpression of *ADH3* improved cryo-tolerance of *S. cerevisiae* but overexpression *GUT2* showed no significant effect.

*Saccharomyces cerevisiae* has a tightly regulated fermentation pathway with a number of alcohol dehydrogenases being glucose repressed, such as *ADH2 and ADH3* (Ciriacy 1975; Young & Pilgrim 1985). *Saccharomyces kudriavzevii* on the other hand does not seem to have such an effective repression mechanism for *ADH3* (Fig.6), which could be due to its preference for the glycerol metabolism, an important pathway associated with cold tolerance (Hayashi & Maeda 2006; Panadero *et al*. 2006).

*Saccharomyces kudriavzevii* and *S. cerevisiae* both have very different glycerol production profiles with *S. kudriavzevii* producing a much larger concentration of glycerol at cold temperatures than *S. cerevisiae* (Arroyo-López *et al*. 2010; Oliveira *et al*. 2014). In the mitochondria, the product of *GUT2* oxidizes glycerol-3-phosphate into dihydroxyacetone, which can enter glycolysis. Therefore, disruption of this gene will hinder glycerol utilization. It is possible that glycerol does not only protect *S. kudriavzevii,* but is also a preferred carbon source at cold temperature. We have performed a preliminary experiment measuring extracellular glycerol produced by *S. kudriavzevii* CA111 *ΔGUT2/ΔGUT2* mutant. Despite the lower fitness of the mutant, we found that there was an accumulation of extracellular glycerol compared with *S. kudriavzevii* CA111, probably due to the inability to break down glycerol as nutrient (Fig. S3, Supporting information).

The most unexpected result of this work was the dramatic increase in fitness at 30 °C of the *S. kudriavzevii GUT2* and *ADH3* mutants when compared to the wild-type strain (Fig.5, Panel A and C). The fitness advantage of the homozygote strains was not as large as their heterozygote counterparts, perhaps because the full removal of the gene may affect several other functions in the cell. As both genes are associated with redox, it could be that their deletion is affecting the strains natural redox balance.

Redox balancing is important for homeostasis within *S. cerevisiae,* and stresses such as ethanol and heat shock are known to disrupt that balance (Piper 1995). During ethanol shock, a much lower concentration of NADH is observed, which indicates that these cells are subjected to a redox imbalance (Vriesekoop *et al*. 2007). Thus, *S. cerevisiae* increases glycerol production during ethanol stress to increase NAD^+^ levels (Vriesekoop *et al*. 2009). Studies have shown that at 38 °C there is a large increase of glycerol-3-phosphate when compared to concentration levels measured at 30 °C (Postmus *et al*. 2008). In heat-shock conditions, it was shown that *S. cerevisiae* produces excess glycerol, a fact exploited by vintners wishing to increase glycerol concentration in wines (Omori *et al*. 1996; Berovic & Herga 2007). These responses could indicate that heat shock, like ethanol stress, may cause a redox imbalance that can be reversed by increasing glycerol production.

The cold acclimation screen highlighted a number of other redox-related genetic knockouts that had significantly decreased or increased fitness, including the two we selected. A number of genes that were haploinsufficient over all three conditions were related to the conversion of reduced NADH or NADPH to the oxidized form, similarly to *ADH3*, for example, mitochondrial 3-oxoacyl-[acyl-carrier-protein] reductase (*OAR1*); diaminohydroxyphoshoribosylaminopyrimidine deaminase (*RIB7*); quinone reductase (*LOT6*). All these mutants may have a decreased NAD^+^ level causing a slower growth rate.

## Conclusions

The use of a genome-scale metabolic reconstruction of yeast metabolism combined with thermodynamic analysis enabled the predictions for metabolic genes associated with cold acclimation. In addition, a screen of the heterozygote yeast deletion collection at 16 °C gave an indication as to which mutations are advantageous or disadvantageous in different media conditions. From these two data sets, *ADH3* and *GUT2* were highlighted as having a strong effect on temperature phenotype and were selected for further genetic studies in different yeast strains and species.

Fitness assays and knockout studies of *ADH3* and *GUT2* mutants suggested that these genes are responsible for the maintenance of the cold-tolerant phenotype. In *S. kudriavzevii* CA111, the disruption of these genes caused a decrease of cryo-tolerance and a dramatic increase of fitness at warm temperature. Both *ADH3* and *GUT2* are more highly expressed in *S. kudriavzevii* CA111 than in *S. cerevisiae 96.2*, and overexpression of *ADH3* in *S. cerevisiae* increases its ability to grow at cold temperature. It can be speculated that these mutants correct a redox imbalance caused by the temperature stress by increasing either glycerol production or cytosolic acetylaldehyde.

Our approach and results provide support for the use of a systems biology framework to studying species adaptation to environmental changes and show that such models can yield testable predictions leading to new biological discoveries.

## Conflict of interests

The authors declare no conflict of interests.

## Abbreviations

D.D. and J.M.S.: conceived and supervised the research.
C.M.P.: carried out both computational and experimental work.
C.M.P., J.M.S. and D.D.: analysed data and wrote the manuscript. All authors read and approved the final manuscript.

## Data accessibility

All required data sets (thermodynamic and genome profiling data) are included in the supporting information.

## Supporting Information

**Figure S1**. Scatter plot of the biological replicates of three time points in different media from the genomewide screen.

**Figure S2.** Growth curve of the overexpression of *GUT2* (panels A and B) and *ADH3* (panels C and D).

**Figure S3.** Extracellular glycerol levels measured at 12 °C and normalized to growth.

**Table S1**. Description of F1, Carbon and Nitrogen limited media.

**Table S2**. List of species specific chimeric knock-out, checking and Real-time primers for *ADH3* (*YMR083w*) and *GUT2* (*YIL155c*) in *S. cerevisiae* and *S. kudriavzevii*.

**Table S3**. List of plasmids used in this study.

**Table S4.** A comparison of ΔG of reactions calculated at 30°C using different thermodynamic equations. R_HEX1 is hexokinase-D glucoseATP; R_SUCRe is sucrose hydrolyzing enzyme; R_ADNK1 is Adenosine kinase and R_RBK is Deoxyribokinase.

**Table S5.** A comparison of ΔG of reactions calculated at 5°C using different thermodynamic equations. R_HEX1 is hexokinase-D glucoseATP; R_SUCRe is sucrose hydrolyzing enzyme; R_ADNK1 is Adenosine kinase and R_RBK is Deoxyribokinase.

**Table S6**. Complete list of ΔG of reactions in the iMM904 *S. cerevisiae* model at standard, 5°C and 30°C.

**Table S7**. Comparison of ΔG of reaction calculated at low and high metabolite concentration for 5°C and 30°C.

**Table S8.** A list of fold change data for the genome profiling carried out at cold temperature.
