# Supplementary figures and images for: Environmental systems biology of cold-tolerant phenotype in Saccharomyces species adapted to grow at different temperatures
Source: Mol Ecol. 2014 Oct 21;23(21):5241–57. doi: 10.1111/mec.12930 (PMC4283049; doi:10.1111/mec.12930)

Figure S1

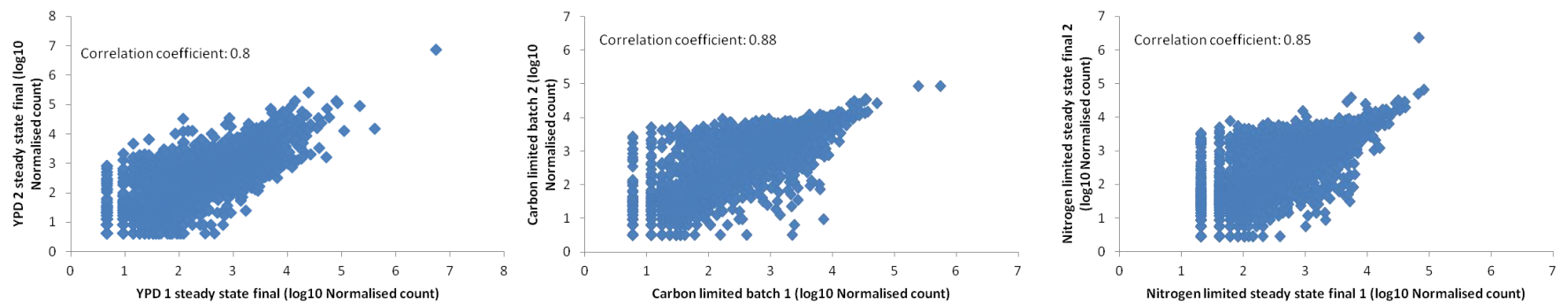

Supplement: Supplementary file 1 — Figure S1. Scatter plot of the biological replicates of three time points in different media from the genomewide screen. [file mec0023-5241-SD1.pdf]

Figure S2

*GUT2*

30°C

A

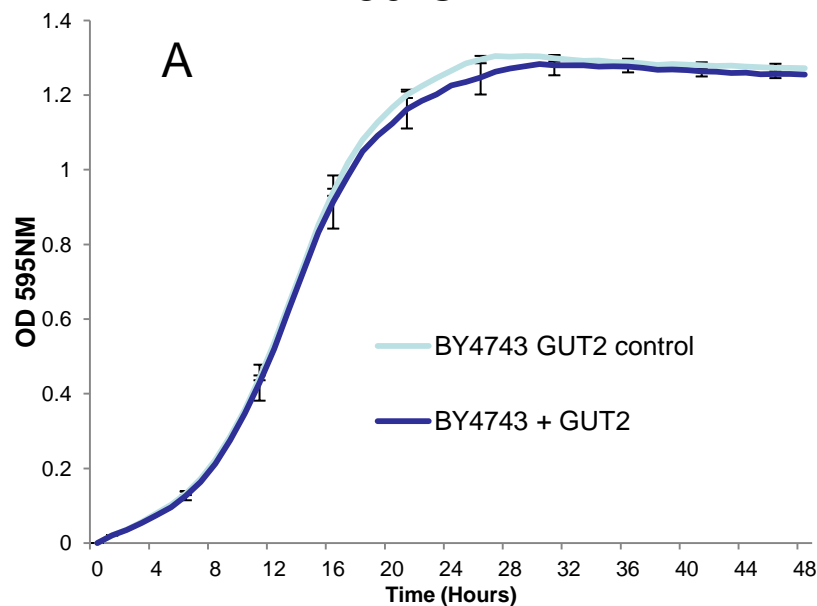

12°C

B

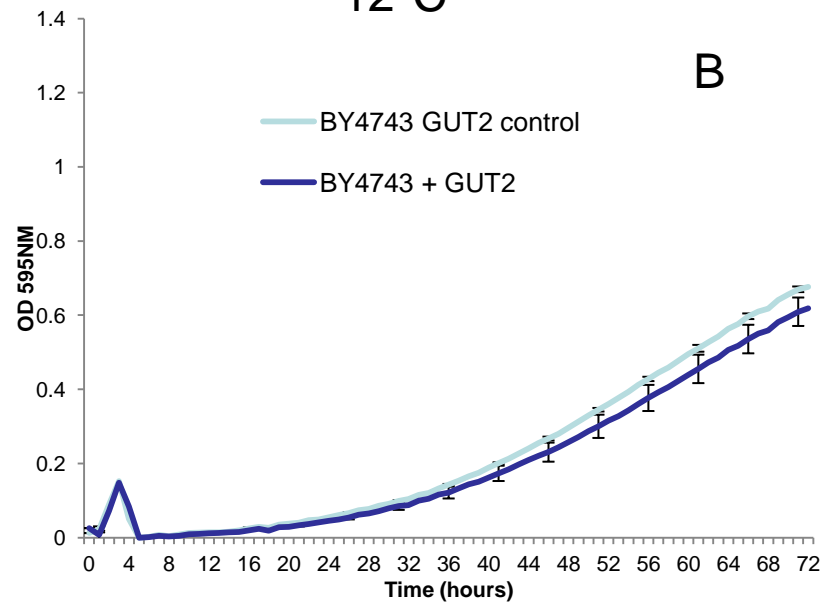

*ADH3*

C

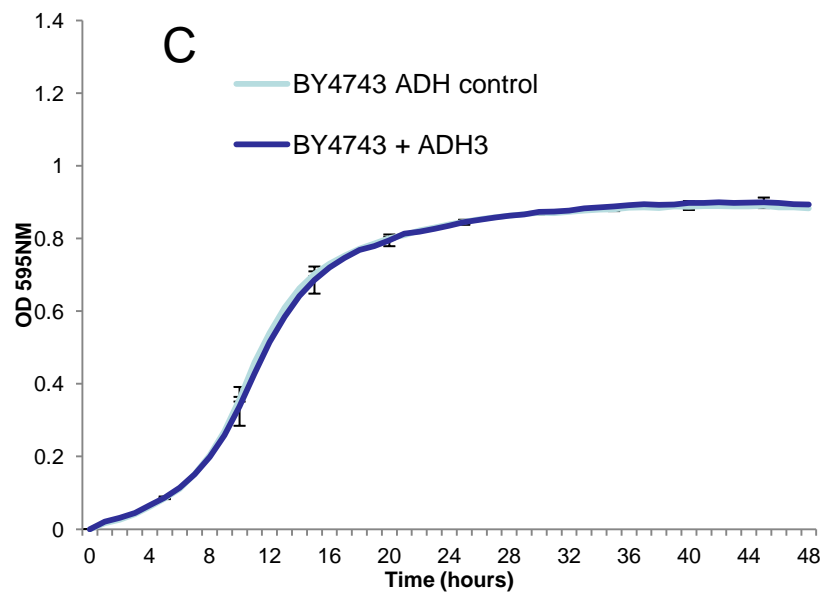

D

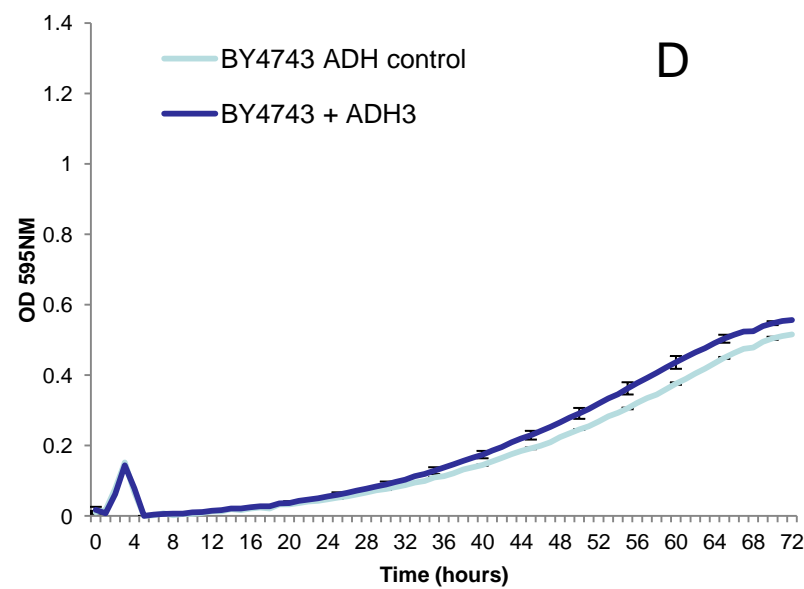

Supplement: Supplementary file 2 — Figure S2. Growth curve of the overexpression of GUT2 (panels A and B) and ADH3 (panels C and D). [file mec0023-5241-SD2.pdf]

Figure S3

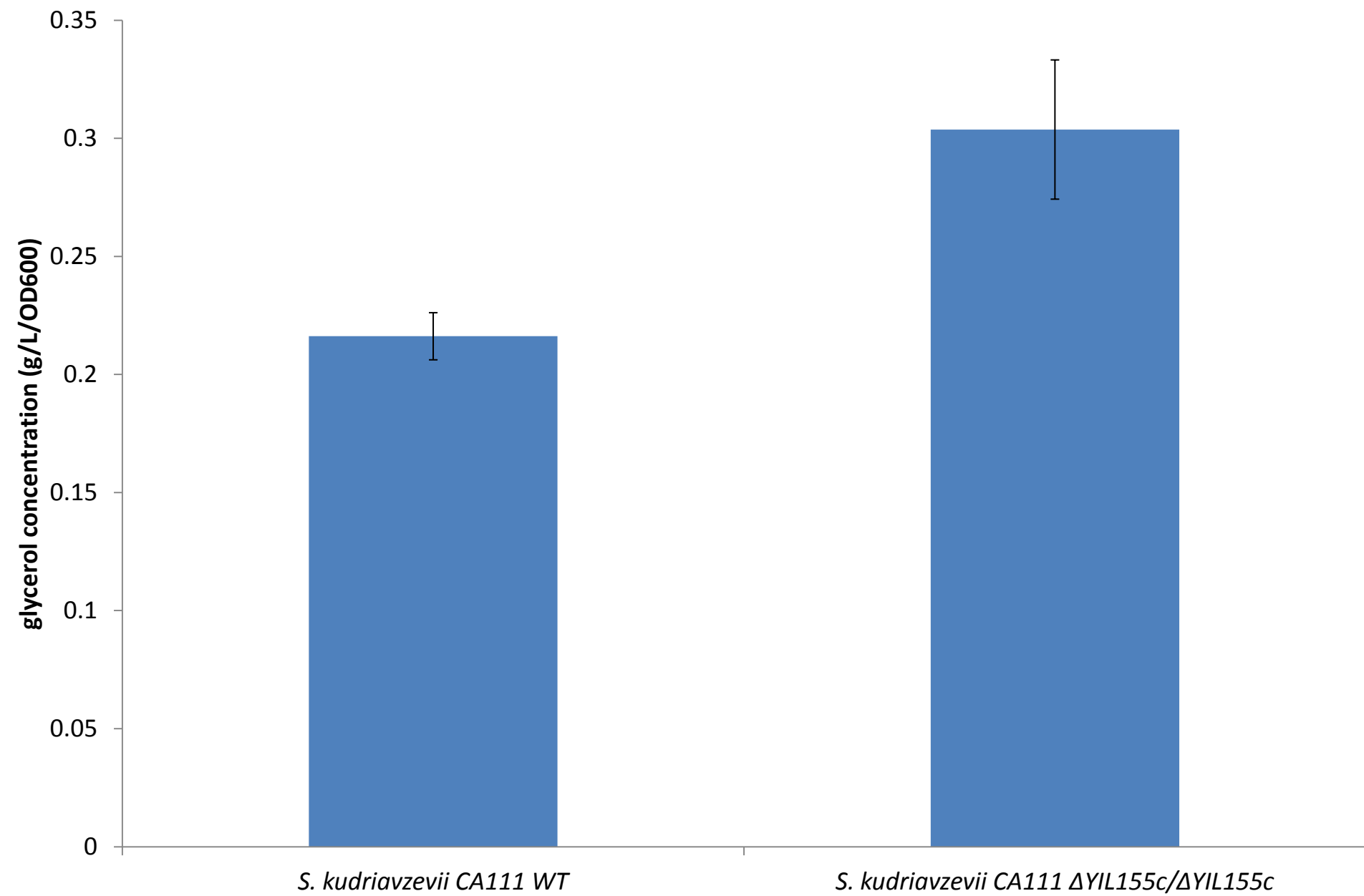

Supplement: Supplementary file 4 — Figure S3. Extracellular glycerol levels measured at 12 °C and normalized to growth. [file mec0023-5241-SD3.pdf]
