# Supplementary material for: Environmental systems biology of cold-tolerant phenotype in Saccharomyces species adapted to grow at different temperatures
Source: Mol Ecol. 2014 Oct 21;23(21):5241–57. doi: 10.1111/mec.12930 (PMC4283049; doi:10.1111/mec.12930)
Supplement: Supplementary file 5 — Table S1. Description of F1, Carbon and Nitrogen limited media. [file mec0023-5241-SD4.docx]

**Table S1: Description of F1, Carbon and Nitrogen limited media**

| Mineral salts solution (Final concentration, g/L) | F1 | F1 Carbon limited | F1 Nitrogen limited |  |
| --- | --- | --- | --- | --- |
| (NH_4_)2SO_4_ | 3.13 | 3.13 | 0.46 |  |
| KH_2_PO_4_ | 2 | 2 | 2 |  |
| MgSO_4_·7H_2_O | 0.55 | 0.55 | 0.55 |  |
| NaCl | 0.1 | 0.1 | 0.1 |  |
| CaCl_2_·2H_2_O | 0.09 | 0.09 | 0.09 |  |
| Glucose | 20 | 2.5 | 20 |  |
|  |  |  |  |  |
| Trace elements solutions |  |  |  |  |
| Trace elements mix 1 | 10,000X stock (g/L) | | Final concentration (mg/L) | |
| ZnSO_4_·7H_2_O | 0.7 |  | 0.07 |  |
| CuSO_4_·5H_2_O | 0.1 |  | 0.01 |  |
| H_3_BO_3_ | 0.1 |  | 0.01 |  |
| KI | 0.1 |  | 0.01 |  |
| – |  |  |  |  |
| Trace elements mix 2^a^ |  |  |  |  |
| FeCl_3_·6H_2_O | 0.5 |  | 0.05 |  |
| Vitamins solution ^b^ |  |  |  |  |
| Vitamins stock solution | 600X stock (g/L) | | Final concentration (mg/L) | |
| Inositol | 37.2 |  | 62 |  |
| Thiamine/HCl | 8.4 |  | 14 |  |
| Pyridoxine | 2.4 |  | 4 |  |
| Ca-pantothenate | 2.4 |  | 4 |  |
| Biotin | 0.18 |  | 0.3 |  |
| ^a^The 10,000X FeCl_3_·6H_2_O stock solution is prepared and kept separately. ^b^The 600X vitamin stock solution is filter-sterilized. | | | | |
