# Supplementary material for: Environmental systems biology of cold-tolerant phenotype in Saccharomyces species adapted to grow at different temperatures
Source: Mol Ecol. 2014 Oct 21;23(21):5241–57. doi: 10.1111/mec.12930 (PMC4283049; doi:10.1111/mec.12930)
Supplement: Supplementary file 6 — Table S2. List of species specific chimeric knock-out, checking and Real-time primers for ADH3 (YMR083w) and GUT2 (YIL155c) in S. cerevisiae and S. kudriavzevii. [file mec0023-5241-SD5.docx]

**Table S2: List of species specific chimeric knock-out, checking and Real-time primers for *ADH3* (*YMR083w*) and *GUT2* (*YIL155c*) in *S. cerevisiae* and *S. kudriavzevii***

| oligoname | sequence |
| --- | --- |
| *S. cerevisiae* *ADH3* upstream chimeric knock-out | TCACAGTTAAAACTAGGAATAGTATAGTCATAAGTTAACACCATCCGTACGCTGCAGGTCGAC |
| *S. cerevisiae* *ADH3* downstream chimeric knock-out | TATAAACAAAGACTTTCATAAAAAGTTTGGGTGCGTAACACGCTAGCATAGGCCACTAGTGGATCTG |
| *S. cerevisiae* *GUT2* upstream chimeric knock-out | CGTGCTATTGCCATCACTGCTACAAGACTAAATACGTACTAATATCGTACGCTGCAGGTCGAC |
| *S. cerevisiae* *GUT2* downstream chimeric knock-out | CTCTTGTGAATGTTATCTTTGTCACCCTTAACTATCATGATCGATGCATAGGCCACTAGTGGATCTG |
| *S. kudriavzevii* *ADH3* upstream chimeric knock-out | CACCATTGTAGGAAACTAGAAAAAGTATATTCATAAAAATTCATCCGTACGCTGCAGGTCGAC |
| *S. kudriavzevii* *ADH3* downstream chimeric knock-out | TACAAACAAAGACTTTTCTATGGAGTCTGCGTGCTTCGCACGCTCGCATAGGCCACTAGTGGATCTG |
| *S. kudriavzevii* *GUT2* upstream chimeric knock-out | AAGAGCTGCTGCTGCTGCTGCTGCCATCGCCACGGCCACAGGATCCGTACGCTGCAGGTCGAC |
| *S. kudriavzevii* *GUT2* downstream chimeric knock-out | TGAAGTTTATAGTTTTTTCAAGTTCCCATTGCCTCTTCTTCTCGGGCATAGGCCACTAGTGGATCTG |
| *S. cerevisiae* *ADH3* inside gene, reverse | TAACAGGTAATGGCCAATCG |
| *S. cerevisiae* *ADH3* outside gene, forward | TTCTGCGTCCGTACACTGTC |
| *S. cerevisiae* *ADH3* inside gene, forward | TCAATATGCAACTGCGATGG |
| *S. cerevisiae* *ADH3* outside gene, reverse | TTCTATGTGTCGACGGAAGC |
| *S. kudriavzevii* *ADH3* inside gene, reverse | GGAATTGCTGCCGTAGATTG |
| *S. kudriavzevii* *ADH3* outside gene, forward | ATCATTCGCTCTCTGCCATC |
| *S. kudriavzevii* *ADH3* inside gene, forward | TCCTACGTTAAGTCGGAGGTC |
| *S. kudriavzevii* *ADH3* outside gene, reverse | GGCTGATTATTAAGAGCACAGG |
| *S. cerevisiae* *GUT2* inside gene, reverse | CTAACGGCCTATCACCTTGG |
| *S. cerevisiae* *GUT2* outside gene, forward | GCAGAACTTCGTCTGCTCTG |
| *S. cerevisiae* *GUT2* inside gene, forward | CAAGATTCGCCTTCTTGGAC |
| *S. cerevisiae* *GUT2* outside gene, reverse | TATGCACCAGGACGTTGAAG |
| *S. kudriavzevii* *GUT2* inside gene, reverse | ACAGGTGAGGAGCCGTATTG |
| *S. kudriavzevii* *GUT2* outside gene, forward | CAGTGAGTAGAAGAAGAGCTGCTG |
| *S. kudriavzevii* *GUT2* inside gene, forward | CTTTCAGGTATCCGTTCACC |
| *S. kudriavzevii* *GUT2* outside gene, reverse | GACACCAAATGTCTTGATGAAG |
| P3K4up kanMX checking primer, reverse | AACGTGAGTCTTTTCCTTACC |
| P2K4down kanMX checking primer,forward | TCGTCACTCATGGTGATTTC |
| real-time S. cerevisiae *ACT*1 Forward | AGA GAT TTG ACT GAC TAC TTG ATG |
| real-time S. cerevisiae *ACT*1 Reverse | GAA GAT TGA GCA GCG GTT TG |
| real-time S. cerevisiae *GUT2* Forward | CGCTAAACGACAACTCCAAG |
| real-time S. cerevisiae *GUT2* Reverse | TCCTTCGGGCAGTAAAAAGA |
| real-time S. cerevisiae *ADH3* Forward | CGACCGCTGATGCTATTC |
| real-time S. cerevisiae *ADH3* Reverse | AAGGAACCCAAGCCACCT |
| real-time S. kudriavzevii *ACT*1 Forward | ATGGTCGGTATGGGTCAAAA |
| real-time S. kudriavzevii *ACT*1 Reverse | GTTCTTCTGGGGCAACTCTCAA |
| real-time S. kudriavzevii *GUT2* Forward | TTCTTTCTACTGCCCGAAGG |
| real-time S. kudriavzevii *GUT2* Reverse | CAAGGGGATGTCTGTGGTG |
| real-time S. kudriavzevii *ADH3* Forward | GTCCCACAGCCTAAACCAAA |
| real-time S. kudriavzevii *ADH3* Reverse | ACGACAACACCAGCACCTTC |
