# Supplementary material for: Environmental systems biology of cold-tolerant phenotype in Saccharomyces species adapted to grow at different temperatures
Source: Mol Ecol. 2014 Oct 21;23(21):5241–57. doi: 10.1111/mec.12930 (PMC4283049; doi:10.1111/mec.12930)
Supplement: Supplementary file 7 — Table S3. List of plasmids used in this study. [file mec0023-5241-SD6.docx]

**Table S3: List of plasmids used in this study**

| Plasmid | Geneotype | Origin |
| --- | --- | --- |
| pBMK630 | pRS315 LEU2 + TDH3 promoter | Ashe Lab, Manchester |
| pBMK631 | pRS316 URA3 + TDH3 promoter | Ashe Lab, Manchester |
| pCMP1 | BMK630 + *GUT2* | This study |
| pCMP2 | BML631 + *ADH3* | This study |
