# Supplementary material for: Environmental systems biology of cold-tolerant phenotype in Saccharomyces species adapted to grow at different temperatures
Source: Mol Ecol. 2014 Oct 21;23(21):5241–57. doi: 10.1111/mec.12930 (PMC4283049; doi:10.1111/mec.12930)
Supplement: Supplementary file 9 — Table S5. A comparison of ΔG of reactions calculated at 5°C using different thermodynamic equations. R_HEX1 is hexokinase-D glucoseATP; R_SUCRe is sucrose hydrolyzing enzyme; R_ADNK1 is Adenosine kinase and R_RBK is Deoxyribokinase. [file mec0023-5241-SD8.docx]

**Table S5: A comparison of ΔG of reactions calculated at 5°C using different thermodynamic equations. R_HEX1 is hexokinase-D glucoseATP; R_SUCRe is sucrose hydrolyzing enzyme; R_ADNK1 is Adenosine kinase and R_RBK is Deoxyribokinase**

| Reaction Name | ΔG calculated in standard conditions (kcalmol^-1^) | ΔG using Gibbs-Helmholtz transformation at 5^o^C (kcalmol^-1^) | ΔG using quotient equation at high concentration at 5^o^C (kcalmol^-1^) | ΔG using quotient equation at low concentration at 5^o^C (kcalmol^-1^) |
| --- | --- | --- | --- | --- |
| R_HEX1 | -14.03 | -13.602 | -12.585 | -17.254 |
| R_SUCRe | -3.113 | -3.476 | -3.113 | -3.113 |
| R_ADNK1 | -14.03 | -13.6 | -14.904 | -15.69 |
| R_RBK | -14.03 | -13.494 | -12.585 | -17.254 |
