# Supplementary material for: Environmental systems biology of cold-tolerant phenotype in Saccharomyces species adapted to grow at different temperatures
Source: Mol Ecol. 2014 Oct 21;23(21):5241–57. doi: 10.1111/mec.12930 (PMC4283049; doi:10.1111/mec.12930)
Supplement: Supplementary file 13 — Data S1. Materials and methods. [file mec0023-5241-SD12.docx]

**Supplementary materials and methods**

**Data analysis of competition experiment**

A Java script was created to extract the tags from the sequencing data. The colorspace sequences were chopped to 19 bp by removing the upstream and downstream universal primers. A synthetic genome was created by concatenating the yeast tags including the flanking universal sequences used as spacers. The tags were then matched to the corresponding gene in the synthetic genome using bowtie ([Langmead *et al.* 2009](#_ENREF_4)). Bowtie was run with the restriction of allowing only two or fewer mismatches.

A further Java program was written to assign gene names to a given tag. The program generated a tab delimited text file for reading into Excel. All the libraries with a minimum number of 1.5 million reads (~250 reads per gene) were analysed (24 out of 28).

Once the count values of each tag were tabulated a weighting score was calculated as per Equation 1. The weighting score was used to normalise all the count values so they are comparable. Each individual data point within a library was multiplied by the libraries weighting score *w_i_*.

**Equation 1**

$$w_{i}=\frac{X}{jx_{i}}$$

Where *w_i_* is the weighting for library *i*, X is the total number of reads from the SOLiD 4 machine, *j* is the number of libraries and *x_i_* is the number of assigned reads for library *i.*

Outlying data points were removed from the data set before averages and fold changes were calculated. To identify outliers each individual strain from a specific condition (pool, YPD, carbon limited and nitrogen limited) was treated as individual data set and converted into a normal distribution with a mean of 0 and a standard deviation of 1 using equation 2. All data points that were further from the mean than the set threshold of 1.1 were deemed outliers. The threshold value of 1.1 was deemed the most suitable as it removed the most likely outliers without losing too much data.

**Equation 2**

$$x'=\frac{x-\overline{x}}{\sigma}$$

Where x’ is the normalised value for x.

Once all outliers had been removed the average was calculated for each time point. Using the averaged data a difference between the initial steady state value and the final steady state value was calculated. The fold changes were converted to log_2_ values.

Strains were deemed haploinsufficient if they had a log_2_ value of less than -0.5. Haploproficient strains were those that had a log_2_ value of more than 0.5.

**Gene Ontology**

Gene Ontology (GO) is a useful tool for analysing which processes or functions are enriched in a list of genes given a background gene list. There are many tools available for Gene Ontology including the Database for Annotation, Visualization and Integrated Discovery (DAVID) ([Huang da *et al.* 2009a](#_ENREF_2), [b](#_ENREF_3)). The advantage of DAVID is that in addition to the large number of up-to-date whole-genome background gene lists that are built-in, there is also the option to upload a customised background gene list. This makes DAVID ideal for GO analysis of genes predicted using metabolic models as the background list can be tailored for the given model. Thus when analysing genes related to the iMM904 model the comparison gene list was changed to the 904 genes found in the model. When analysing data from the genome-wide cold phenotype screen DAVID was run using the standard *S. cerevisiae* background gene list.
